# The complete chloroplast genome of a medicinal plant, *Wikstroemia chamaedaphne* (Thymelaeaceae)

**DOI:** 10.1080/23802359.2019.1711228

**Published:** 2020-01-16

**Authors:** Shao-Juan Qian, Yong-Hong Zhang, Guo-Dong Li

**Affiliations:** a School of Life Sciences, Yunnan Normal University, Kunming, China;; b Faculty of Traditional Chinese Pharmacy, Yunnan University of Chinese Medicine, Kunming, China

**Keywords:** *Wikstroemia chamaedaphne*, chloroplast genome, medicinal plant, phylogenetic analysis

## Abstract

The first complete chloroplast genome of *Wikstroemia chamaedaphne*, a poisonous shrub with important medicinal value, is reported in this study. The plastome is a quadripartite circular shape with 173,042 bp in length. It consists of a large single-copy (LSC) region of 86,330 bp and a small single-copy (SSC) region of 2868 bp, separated by two inverted repeat (IR) regions of 41,922 bp each. The chloroplast genome contains 137 genes, including 91 protein-coding genes, 38 tRNA genes, and 8 rRNA genes. The GC content values in the whole cp genome, LSC region, SSC region, and IR region are 36.6%, 34.6%, 28.3%, and 38.9%, respectively. The corresponding numbers of mono-, di-, tri-, tetra- and penta-nucleotides SSRs were 73, 13, 9, 13, and 1. Phylogenetic study revealed that *W. chamaedaphne* and *W. indica* formed a monophyletic branch and having a close relationship with *Stellera chamaejasme*.

*Wikstroemia chamaedaphne* Meisn., a poisonous shrub belonging to the genus *Wikstroemia* (Thymelaeaceae), has been used in traditional Chinese medicines to treat cough, edema, schizophrenia, hepatitis, and antifertility (Yu et al. 2002; Guo et al. 2015). Previous studies on *W. chamaedaphne* are mainly focused on the separation, extraction, and clinical application of active components (Guo et al. 2015; Li et al. 2018). Until now, no genomic information on *W. chamaedaphne* has been reported. In this study, the chloroplast genome of this species and its phylogenetic position were studied.

The fresh leaves of *W. chamaedaphne* were collected from a healthy plant located at Yaodu district, Linfen city (36°07′34.39″N, 111°18′26.03″E), Shanxi Province, China. The voucher specimens (ZYZ-20140628) were deposited in the Herbarium of Yunnan Normal University. A sequence library was constructed and sequencing was performed using the Illumina HiSeq 2500-PE150 platform (Illumina, San Diego, CA, USA). All raw reads were filtered to obtain clean reads with default parameter using NGS QC Toolkit_v2.3.3 (Patel and Jain 2012). The plastome was de novo assembled using NOVOPlasty (Dierckxsens et al. 2017) and annotated using Geneious 9.1 (Kearse et al. 2012).

The complete cp genome of *W. chamaedaphne* (Genbank accession no.: MN563132) is quadripartite circular shape with 173,042 bp in length, comprising a large single-copy region (LSC) of 86,330 bp and a small single-copy region (SSC) of 2868 bp, separated by a pair of inverted repeat (IR) regions of 41,922 bp. The GC content of the total genome is 36.6%, and the IR regions have a higher GC content (38.9%) than LSC (34.6%) and SSC (28.3%). The cp genome encoded 137 genes, including 91 protein-coding genes, 38 transfer RNA genes and 8ribosomal RNA genes. IMEx was used to identify SSRs with the minimum repeat number set to 10, 5, 4, 3, 3, and 3 for SSRs (Mudunuri and Nagarajaram 2007). The numbers of mono-, di-, tri-, tetra- and penta-nucleotides SSRs were 73, 13, 9, 13, and 1, respectively.

To determine the phylogenetic position of *W*. *chamaedaphne* with respect to the other species of Thymelaeaceae, the reported complete chloroplast genomes of eight species of Thymelaeaceae, five species of Malvaceae, and three outgroups were used to build the phylogenetic tree. All sequences were aligned by MAFFT 7.308 (Katoh and Standley 2013) and the maximum likelihood (ML) tree was reconstructed by RAxML 8.2.11 (Stamatakis et al. 2008) with the nucleotide substitution model of GTR + G. Bootstrap values were calculated from 1000 replicates analysis. The phylogenetic analysis revealed that all sampled species of Thymelaeaceae were clustered into one clade with 100% bootstrap value. Within Thymelaeaceae, *W. chamaedaphne* and *W. indica* formed a monophyletic branch closing to *Stellera chamaejasme* with 100% support. The *Wikstroemia – Stellera* clade and the clade of three *Daphne* species formed sister groups with 100% support (Figure 1). The current phylogenetic analysis will be useful for studying the phylogeny of *Wikstroemia* in the future.

**Figure 1.**
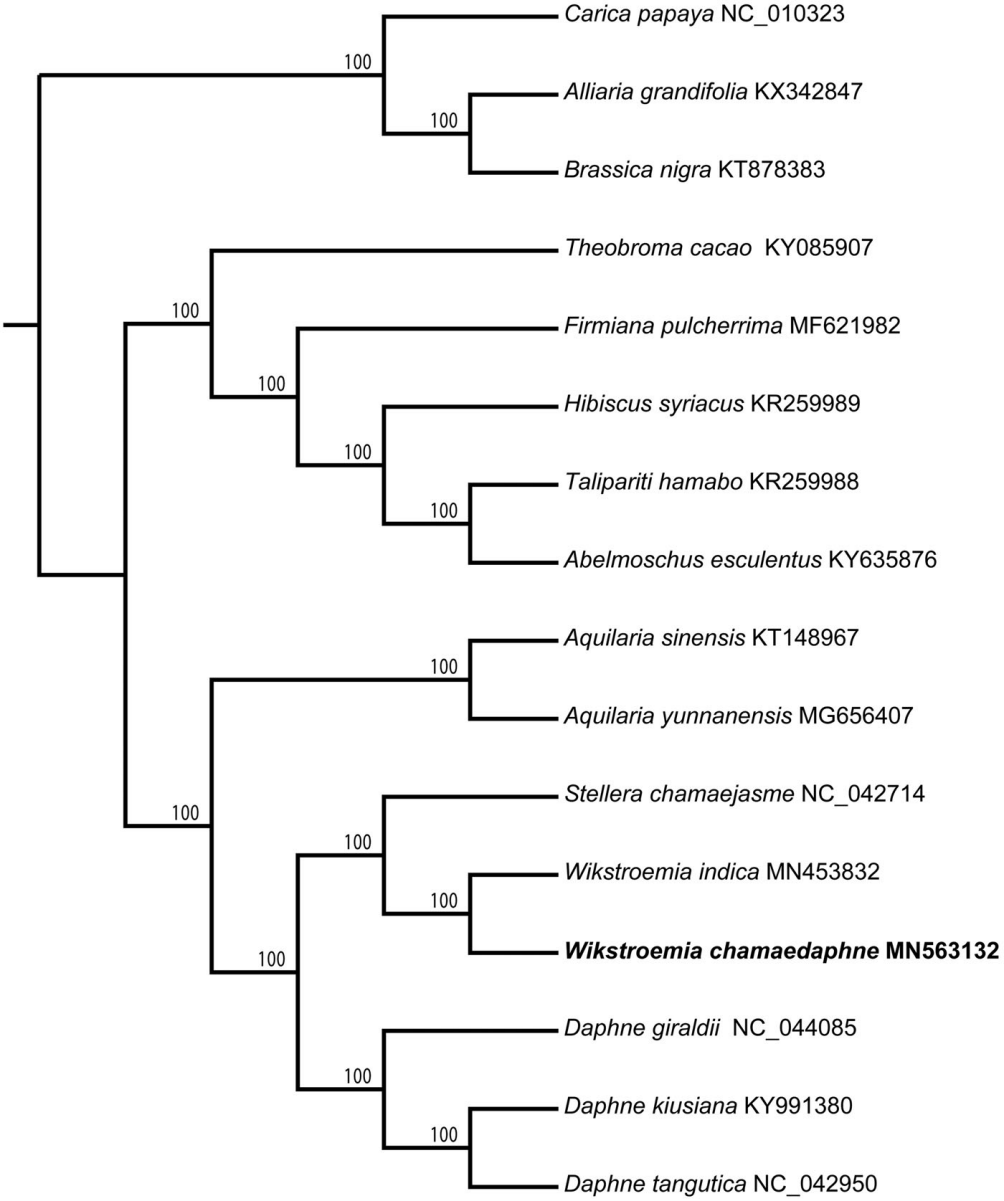
Maximum-likelihood (ML) tree of *W. chamaedaphne* and its related relatives based on the complete chloroplast genome sequences. Bootstrap values from 1000 replicates were shown next to the nodes.
